# The Importance of Anharmonicity and Solvent Effects on the OH Radical Attack on Nucleobases

**DOI:** 10.3390/ijms25063118

**Published:** 2024-03-08

**Authors:** Anna Thorn Ekstrøm, Vera Staun Hansen, Stephan P. A. Sauer

**Affiliations:** Department of Chemistry, University of Copenhagen, 2100 Copenhagen, Denmark; ate@chem.ku.dk (A.T.E.);

**Keywords:** DNA damage, OH-radicals, DFT calculations, radiation therapy

## Abstract

Previous theoretical investigations of the reactions between an OH radical and a nucleobase have stated the most important pathways to be the C5-C6 addition for pyrimidines and the C8 addition for purines. Furthermore, the abstraction of a methyl hydrogen from thymine has also been proven an important pathway. The conclusions were based solely on gas-phase calculations and harmonic vibrational frequencies. In this paper, we supplement the calculations by applying solvent corrections through the polarizable continuum model (PCM) solvent model and applying anharmonicity in order to determine the importance of anharmonicity and solvent effects. Density functional theory (DFT) at the ωB97-D/6-311++G(2df,2pd) level with the Eckart tunneling correction is used. The total reaction rate constants are found to be 1.48 $\times \;10^{-13}$ cm^3^ molecules^−1^s^−1^ for adenine, 1.02 $\times \;10^{-11}$ cm^3^ molecules^−1^s^−1^ for guanine, 5.52 $\times \;10^{-13}$ cm^3^ molecules^−1^s^−1^ for thymine, 1.47 $\times \;10^{-13}$ cm^3^ molecules^−1^s^−1^ for cytosine and 7.59 $\times \;10^{-14}$ cm^3^ molecules^−1^s^−1^ for uracil. These rates are found to be approximately two orders of magnitude larger than experimental values. We find that the tendencies observed for preferred pathways for reactions calculated in a solvent are comparable to the preferred pathways for reactions calculated in gas phase. We conclude that applying a solvent has a larger impact on more parameters compared to the inclusion of anharmonicity. For some reactions the inclusion of anharmonicity has no effect, whereas for others it does impact the energetics.

## 1. Introduction

Worldwide, roughly 10 million people die from cancer every year, which makes it the second-leading cause of death after heart diseases [1]. Radiation treatment remains one of the most-used treatments for cancer and even after years of research on cancer treatments, radiation treatment still also exposes healthy cells to the radiation; thus, the Deoxyribonucleic acid (DNA) in the healthy cells is damaged as a consequence of the treatment. Consequently, improved methods for radiation treatment such as hadron therapy are relevant [2].

**Figure 1:**
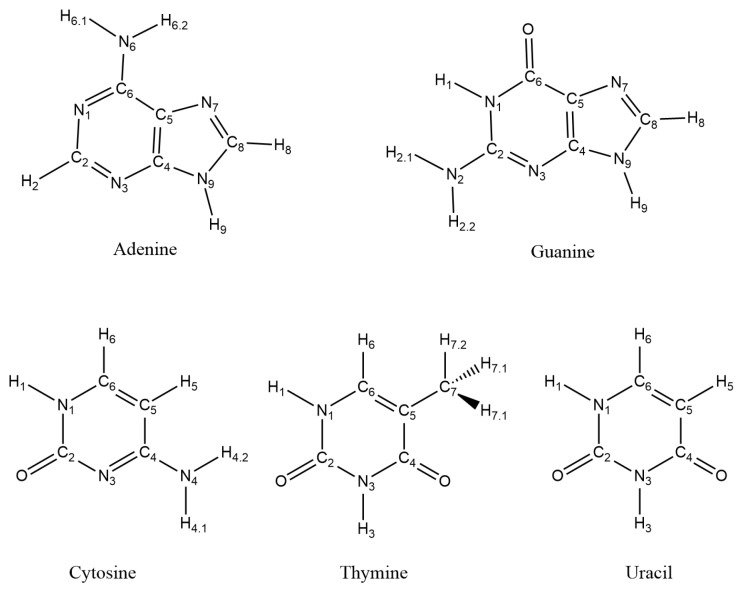
Numbering scheme for the nucleobases.

Radiation treatments work by exposing infected cells to high-energy radiation or particles to destroy the DNA and thereby prevent the diseased cells from dividing further [3]. Thus, the radiation of cancer cells leads to two different outcomes: the first one is a positive effect, which consists of a decrease in the rate of division of cancer cells, and the second one is the possible damage of DNA. Either direct radiolysis of DNA is observed or the DNA string is indirectly damaged by radicals generated in the cell by the radiation. The cells in a human body consist mainly of water (70 percent). The radiolysis of water and how it affects DNA is thus of great importance, as the probability of the radiation hitting a water molecule is not negligible [4]. A radiated water molecule decomposes into highly reactive radical species, such as an OH radical [5], and this can cause a reaction with the DNA in the cells, leading to mutations, severe damage of the DNA or the death of the cell. The OH radical can undergo two different reactions with the nucleobases of DNA: a hydrogen abstraction reaction or an addition reaction where the radical adds to the double bonds in the aromatic nucleobases. Both reactions lead to nucleobase radicals, which are highly reactive and eventually lead to strand breaks within the DNA, resulting in mutation in the cell or cell death [6,7]. The nucleobases consist of two purines: adenine and guanine, and three pyrimidines: thymine, cytosine and uracil (found in Ribonucleic Acid (RNA)); see Figure 1. In addition, hydrogen abstraction from the ribofuranose of the nucleosides are possible [8]. Even though the OH radical reacts aggressively with organic compounds, it is short-lived; thus, to react with DNA the OH radical must be adjacent to the DNA string [9]. Experiments have shown that the indirect reaction through water occurs 60–70 percent of the time, which is why it is of great importance to improve knowledge about such reactions [4].

In the present study, we have investigated by density functional theory (DFT) [10,11,12] calculations how water as solvent impacts the abstraction and addition reactions that all five different nucleobases found in RNA or DNA can undergo. Furthermore, we have included anharmonic corrections to the vibrational frequencies in the calculation of the partition functions and free energies in order to see if it impacts the reaction rates, since some of the vibrational modes for the transition states are likely not to be harmonic. The total reaction rate constant will be discussed for all nucleobases and compared to gas-phase or harmonic results and experimental data.

Previous work by our group [13,14] has investigated the relevant reactions for the five nucleobases at the DFT level employing the ωB97X-D exchange-correlation functional [15] in combination with Pople’s triple-zeta 6-311++G(2df,2pd) basis set [16] and the Eckart tunneling correction [17]. However, all calculations were carried out in gas phase, which might be misleading compared to the environment in the human body where these reactions occur. Also, the calculations were carried out under the assumption that the frequencies are harmonic. The following conclusions were drawn in the previous study [14]. For both guanine and adenine, every abstraction apart from H8 seems likely. H8 is unfavorable since a large activation barrier is observed and only a small amount of energy is gained. Milhøj and Sauer, however, state that abstraction from amine groups seems most plausible (H6.1, H6.2). For guanine, H2.1 is found to have the lowest Gibbs free energy and these abstractions are therefore considered more likely. For adenine, addition onto any carbon except C6 seems likely; however, the possibility of the addition depends on the degree to which the reaction breaks the aromaticity of the ring. Thus, C8 and C2 are the favored addition sites and C6 is the least likely. Addition onto the nitrogen atoms is unimportant since the energy barriers are large compared to C-addition. For guanine, the trends observed are similar, where the C8 and C5 additions seem plausible and C6 seems unlikely. In the three pyrimidine nucleobases, the same tendencies are observed. Through an examination of the highest occupied molecular orbitals (HOMO), the addition onto the C5-C6 bond is expected for all three pyrimidine nucleobases. This is due to the high electron density around this bond. For thymine, the abstraction of the methyl hydrogens is favored with an activation barrier of 21 kJ/mol; for cytosine, all Gibbs free energies are close (around 38–45 kJ/mol), but the smallest barrier is found for H4.2. For uracil, the abstraction of H1 is found to be most likely; however, since the sugar in DNA is placed at this bond, this is not an abstraction that can happen in DNA. It was also concluded that the H6 abstraction even with a somewhat larger barrier is still plausible. The overall reaction rates, when applying the Eckart tunneling factor, are for adenine, $1.06\times 10^{-12}$; guanine, $2.31\times 10^{-11}$; cytosine, $8.50\times 10^{-13}$; thymine, $1.95\times 10^{-11}$; and uracil, $1.89\times 10^{-13}$ cm^3^ molecules^−1^s^−1^ [14]. In general, this study concludes that both abstraction and addition reaction pathways are important to determine an overall reaction rate.

Later, the influence of different solvent models (PCM [18,19], explicit microsolvation modeling of a single and two water molecules and a combination of both) on the calculations of these reactions is studied for adenine [20] and thymine [21]. Based on the calculations performed within the earlier developed computational protocol, Sørensen and Sauer [21] have concluded that, for the abstraction of hydrogen in the methyl group of thymine, the PCM solvent model appears to be the better choice due to a better agreement of the calculated rate constants with their experimental values. For adenine, which does not have a corresponding methyl group, Milhøj and Sauer [20] have studied not only all hydrogen abstraction reactions but also the OH addition reactions. They observed that for some reactions the PCM model and explicit microsolvation lead to opposing conclusions about the solvent effects, as was observed for the hydrogen abstraction from the methyl group in thymine, while for other reactions both models lead to the same conclusions. This is probably not surprising, if one considers the significant differences between abstraction and addition reactions in the electronic environment of the various reaction sites in adenine.

Similar research has been carried out by Ji et al. [22], where the addition and abstraction reactions between the OH radical and cytosine were investigated, including solvation by single-point B3LYP/6-31++G(d,p)//B3LYP/6-31G(d,p) conductor-like polarizable continuum model (CPCM) calculation [23,24,25,26]. Based on their research, it was concluded that the addition to C5 and C6 is preferred and occurs most. The results showed that in a thermodynamic aspect the addition to C6 or C5 is preferred, because it is more thermodynamically favorable, and, in the kinetic aspect, the addition to C6 or C5 is preferred due to a very small barrier for C6 and practically next to negligible barrier for C5. The agreement between the calculated and experimental data corroborates the theoretically established fact that the C5 addition is preferred. This suggested the reaction to be kinetically controlled. For the abstraction reactions, it was found that the H4.2 has the weakest bond and the smallest barrier, which makes it a favorable hydrogen to abstract both kinetically and thermodynamically. They observed a rather high probability of addition onto C5 compared to the abstraction. These reactions were also carried out in the gas phase, but similar tendencies were observed in an aqueous medium. Overall, the OH radicals react with cytosine mainly by addition onto C5 and C6. As a main result, Ji et al. [22] proved that applying a solvent model does not alter the relative reaction pathways, but the energy barriers vary significantly.

For uracil, the reactions have been investigated by Prasanthkumar et al. [27] using B3LYP/6-31+G(d,p) and MPW1K/6-31+G(d,p) [28] and applying the PCM model in the geometry optimization. The energy was further improved by applying CCSD(T)/6-31+G(d,p) [29] single-point corrections. They argued that due to the double-bond character for the C5-C6 bond and thereby higher electron density, an electrophile reaction such as the OH addition would prefer this specific bond. Both addition reactions to either C5 or C6 were almost barrierless with activation energies of 0.4 kcal/mol (C5) and 0.2 kcal/mol (C6). The small difference in activation barrier might nevertheless cause regioselectivity. In a thermodynamic perspective, UC6OH was a more stable compound and, therefore, the favored addition product. Applying a PCM model influenced the destabilization slightly, but the tendencies for the energies and kinetics were conserved. For the abstraction reaction, it was found that H1 is favored kinetically, but again impossible in DNA due to the sugar linkage.

Kakkar and Garg [30] analyzed the OH reaction with thymine using the AM1 method [31] within the MOPAC 7.0 program [32]. Both restricted Hartree–Fock and unrestricted Hartree–Fock methods were used for the geometry optimization for the radicals. They found that for thymine the C5 and C6 additions and the methyl group abstraction are important for the overall reaction. For thymine, Ji et al. [33] employed the DFT method with the B3LYP function. In order to obtain molecular geometries, the basis set 6-31++G(d,p) was employed for single-point energy calculations at B3LYP/6-31G(d,p) optimized structures. They found results in agreement with those of our group. For additions, the C5 and C6 carbons are preferred due to the small to no energy barriers. For C6, no activation energy barrier is observed and for C5, the barrier is 0.70 kcal/mol. For abstraction, the methyl hydrogens are preferred with a slightly higher activation barrier at 1.88 kcal/mol. The results are in agreement with experiential values. The article states that C6 is more energetically favorable in comparison to C5 also when solvent effects are accounted for. The article states furthermore that the C5 addition reaction occurs 30 percent of the time, whereas the C6 addition occurs 60 percent of the time and methyl abstraction occurs 10 percent of the time, in contrast to the experimental values: 60 percent, 35 percent and 5 percent, respectively.

For the purines, Kumar et al. [34] investigated the reaction between the OH radical and guanine in an aqueous environment using DFT calculations. The interaction between the nucleobase and the radical has been investigated both in gas phase and by employing the single-point PCM solvent model or in combination with 12 explicit water molecules using the B3LYP/6-31G* method. The reaction between the radical and guanine with 12 water molecules applied has been explored with the B3LYP functional and the 6-31G* or 6-31++G** basis sets. Frequency calculations were, however, not performed to verify true transition states. The study states that both theory and experiments on similar structures conclude the abstraction reactions and addition reactions should be two competitive pathways. The study showed that the energy for the initial binding of an OH radical with guanine in a hydrated environment follows the order C8 > C4 > C5 > N1 abstraction. These are similar tendencies as for the gas phase. A stabilization of the N1 heterocyclic nitrogen was observed when applying the PCM model. This suggests that N1 abstraction would be favored.

In 2010, Cheng et al. [35] showed the importance of the different dehydrogenating reactions for adenine in a study concerning the hydroxyl reaction with adenine employing B3LYP/DZP++ level of theory. Optimized geometries, harmonic vibrational frequencies, natural populations and intrinsic reaction coordinate (IRC) calculations were performed. The study states that, except for C8 radical attack, the hydroxyl attacks all other sites that lead to dehydrogenation, spontaneously forming the adenine radical and water. H6.2 was found to be the most kinetically favorable.

It should be noted that the thermodynamic aspects of these reactions and conclusions based upon thermodynamics are only slightly relevant, since the products are highly reactive and will likely react further with oxidants or other molecules in the environment. This has been investigated in multiple studies, and Naumov and von Sonntag [36] stated that reactions between the OH radical and nucleobases are likely to be kinetically controlled rather than by thermodynamics, since thermodynamic equilibrium is not upheld and thus, they undergo tautomerization reactions, water elimination reactions and rearrangement reactions.

Asmus et al. [37] also investigated the radiolysis of pyrimidine and purines, presenting the results of studies of reactions of organic free radical products produced by OH radical attack on nucleobases. They stated that a free nucleobase can subsequently react with any sensitizer present in the environment, resulting in an electron transfer process or an addition, which can lead to fixation of the radiation damage. Different experimental approaches have been carried out in order to determine specific radiation reactions.

Corresponding experimental studies [38,39,40] have investigated the reaction pathways between the nucleobases and an OH radical. In these studies, it is supported that for the pyrimidines the C5/C6 addition is the dominant reaction pathway. In the following studies, the reaction pathways of the produced radicals with different compounds have been investigated, and this supports that thermodynamic equilibrium is not upheld. Based on experiments, the comparison between the addition pathway and the abstraction pathway for cytosine clearly states that the addition reaction is expected more than 80 percent of the time, and the addition on the C5/C6 double bond is clearly the most dominant. Of C5 and C6, the C5 addition is preferred 87 percent of the time [38]. This tendency also applies to thymine and uracil, where addition of the OH onto the double bond is also clearly favored, but abstraction from the methyl group in thymine also plays an important role [39]. For all three reactions, there is experimental evidence that the radicals formed are reactive and will react further. This is in good agreement with the already-discussed theoretical conclusions. For the purines, addition is also the primary reaction pathway, and C8 is the most dominant addition, but other relevant additions such as C5 and C4 have also been observed, resulting in the formation of three different radicals [40].

Based on the previous studies, it is not clear what the effect is of applying PCM to the reaction between a hydroxyl and a nucleobase. As a consequence of different treatment of the various nucleobases in previous investigations and of many different computational approaches employed, it is difficult to directly draw conclusions on the importance and influence of PCM in such reactions. Furthermore, to our knowledge, previous studies have only considered harmonic frequencies in the calculation of free energies or rate constants.

Therefore, this study will investigate the importance of applying the PCM solvent model and anharmonicity in all steps of the calculations (geometry optimizations and frequency calculations) of the different individual reaction pathways within all five nucleobases. Thus, this study specifically investigates how solvent effects and anharmonic frequencies might impact the stability of different species, reaction barriers and consequently, the rate constants for the addition and abstraction reactions, including both a kinetic and thermodynamic point of view.

## 2. Results and Discussion

The results obtained in this study will be compared to the experimental values of Scholes, who determined the absolute rate constants for uracil, thymine, cytosine, adenine and guanine to be $7.47\times 10^{-12}$, $6.44\times 10^{-12}$, $6.97\times 10^{-12}$, $7.14\times 10^{-12}$ and $1.53\times 10^{-12}$ cm^3^ molecules^−1^s^−1^, respectively [41].

The tendencies observed within the purines generally show similar behavior for different reaction pathways due to similarities in the chemical structure. The pyrimidines share a similar chemical structure as well; thus, they are expected to show similar tendencies.

The inclusion of anharmonicity was tested for one of the pyrimidines, uracil, and one of the purines, adenine. These results are expected to be transferable to the general picture of the importance of anharmonicity, since it is expected that the properties observed for one of these structures can be transferred to the others.

### 2.1. Testing for Solvent Effects and Anharmonicity

In order to determine the importance of anharmonicity and solvent effects, three different calculations were made for each reaction with uracil and adenine. For every predicted abstraction or addition pathway, one calculation including anharmonicity in gas phase, one calculation including the water solvent and one calculation including anharmonicity in an aqueous environment were carried out. For each of these calculations, the Eckart tunneling factor and the reaction rate constants were calculated. These results are, furthermore, compared to the gas-phase result for similar pathways found by our group [14]. This was conducted in order to determine whether it is a fair assumption to make the calculations in gas phase and to determine if it is valid to carry out calculations without including anharmonicity.

#### 2.1.1. Uracil

Table 1 and Table 2 show the reaction rate constants with the Eckart tunneling factor and imaginary frequency in parentheses for every possible reaction pathway within uracil for harmonic frequencies in gas phase [14], anharmonic frequencies in gas phase, applying water as a solvent with harmonic frequencies and a combination of anharmonic frequencies and applying water as a solvent.

In general, there is a noticeable tendency for the Eckart tunneling factor for the anharmonic and harmonic calculations in the gas phase to be very similar. This goes for both abstraction and addition reactions, and it implies that applying the anharmonic frequency calculation does not really affect the tunneling factor.

On inclusion of water through the PCM solvent model, an increase in the tunneling factor is observed for abstraction pathways. For every abstraction, a larger tunneling factor is observed, but especially for H3, the tunneling factor is affected significantly by the solvent. For the addition reactions, the tunneling factors are more similar and not particularly affected by applying a solvent model. However, in general, the tunneling factor has a smaller impact on addition reactions compared to abstraction reactions where tunneling is more common.

Calculations including both anharmonic and solvent corrections show an increase in the tunneling factor for every abstraction reaction compared to the gas-phase results. For both additions and abstractions, the anharmonic calculations with PCM are found to be in better agreement with the harmonic calculations with PCM. This emphasizes that anharmonic frequencies have no noticeable impact on the tunneling factor.

For the abstraction reactions, the tunneling factors are more comparable for calculations in gas phase and more comparable for calculations in solvent. Similarly, especially the addition reactions show agreement between the tunneling factors for the two different solvent calculations and the two different gas-phase calculations. This indicates that the anharmonicity has no important impact on the tunneling factors.

The imaginary frequency for each transition state was investigated and the results correspond to the already-observed tendencies. Thus, the imaginary frequencies for the harmonic and anharmonic gas-phase calculations are close, and the imaginary frequencies for the harmonic and anharmonic PCM calculation are almost identical and mostly higher than those from the gas-phase calculations. This shows that the results of anharmonic calculations do not differ much from the harmonic calculations, underlining that anharmonicity has a negligible impact. Applying a solvent impacts both the tunneling factor and the imaginary frequencies significantly. Similarly, the frequency from the anharmonic PCM calculation does not differ much from the one from the harmonic PCM calculations. Thus, the anharmonic corrections to the frequencies can presumably be neglected, while the solvent has a noticeable impact.

**Figure 2:**
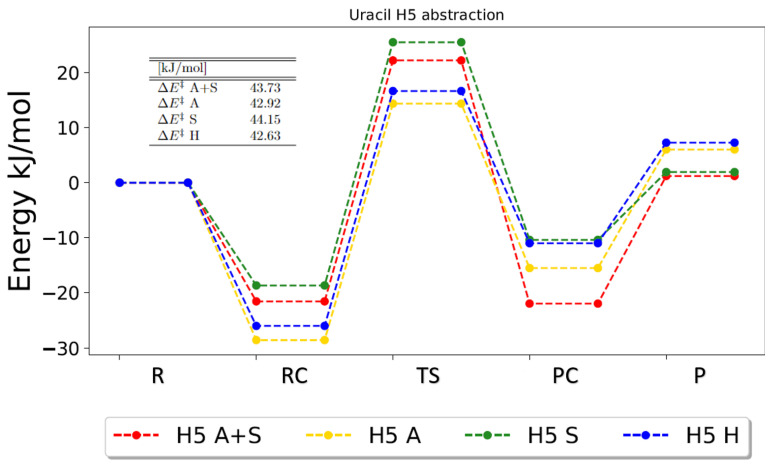
Zero-point vibrational corrected energies for the abstraction of H5 in uracil. A+S indicates anharmonic PCM calculation, A indicates anharmonic gas-phase calculations, S indicates harmonic PCM calculations and H indicates harmonic gas-phase calculations. R, RC, TS, PC and P denote the reactant, the reactant complex, the transition state, the product complex and the products, respectively.

In order to comment on the impact on energetics, potential energy surfaces (PESs) for every reaction pathway and their corresponding zero-point vibrational corrected energies were obtained. These PESs underline the already-discussed tendencies, i.e., that similarities between the gas-phase calculations were observed as well as similarities between the solvent calculations. For every abstraction reaction, the energetics for the gas-phase calculations seem to be in best agreement with each other, and the energetics for the solvent calculations agree best. This is illustrated for the abstraction of H5 in Figure 2, where the energetics for gas-phase calculations are similar, and the energetics for PCM calculations are similar. This is the general picture observed for abstractions in uracil, as can be seen from the figures for the other abstraction reactions in Appendix A.

**Figure 3:**
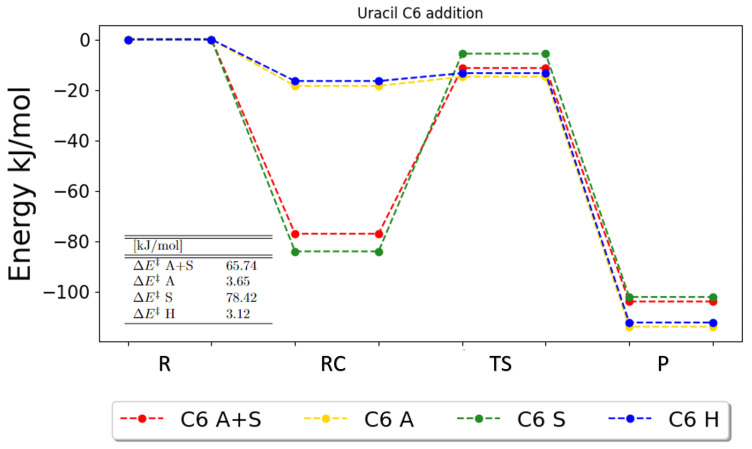
Zero-point vibrational corrected energies for the addition onto C6 in uracil. A+S indicates anharmonic PCM calculation, A indicates anharmonic gas-phase calculations, S indicates harmonic PCM calculations and H indicates harmonic gas-phase calculations. R, RC, TS, PC and P denote the reactant, the reactant complex, the transition state, the product complex and the products, respectively.

For the addition reactions, the same pattern is observed, as illustrated in Figure 3 for the addition to C6. This is also the general picture of the trend for the other addition reactions in uracil, for which the figures can be found in Appendix A. Furthermore, it should be noted that the solvent calculations have a higher forward barrier compared to the gas-phase calculations; thus, a lower reaction rate is to be expected.

Finally, the reaction rates for the abstraction pathways show the same trends as already discussed for the tunneling factor, the imaginary frequency and the zero-point vibrational corrected energies. The reaction rates for gas-phase harmonic and anharmonic calculations are, in general, in good agreement with each other. On the other hand, when applying the solvent model and when applying the combination of anharmonic and solvent corrections, the total rate constant is, in general, observed to be smaller. This underlines the noticeable correlation between the two gas-phase calculations and the correlations between the solvent calculations and shows again that inclusion of the anharmonic corrections has the least important effect, while inclusion of solvent has an important influence. From this, we can verify that it to some extent would be a fair assumption to ignore the anharmonic effects for uracil. On the other hand, the PCM solvent model affects the reaction rates vastly and cannot be ignored.

#### 2.1.2. Adenine

Similar calculations were carried out for adenine to verify whether we also find the same trends for the bigger nucleobases. Table 3 and Table 4 show the reaction rate constants with the Eckart tunneling factor and imaginary frequencies in parentheses for every possible reaction pathway within adenine. They were calculated again as harmonic frequencies in gas phase [14], anharmonic frequencies in gas phase, applying water as a solvent with harmonic frequencies and a combination of anharmonic frequencies when applying water as a solvent. Table 3 and Table 4 for adenine will in the following be discussed to determine the impact of modeling the solvent and the anharmonicity of the frequencies.

The Eckart correction factors for the abstraction reactions show to some extent an agreement between the tunneling factors for harmonic and anharmonic PCM calculations (see Table 3). Furthermore, an agreement between harmonic and anharmonic gas-phase calculations can be observed. This is, in particular, seen for the abstraction of H6.2, where the tunneling factors from harmonic and anharmonic PCM calculations are very large, whereas the gas-phase results are noticeably smaller. For the abstraction of H8, the gas-phase results are observed by their extremely large tunneling factor (to the ninth power), whereas the harmonic PCM is much smaller and for the anharmonic PCM calculations the tunneling factor cannot even be calculated due to a change in the balance of energetics and thus, negative barriers.

For the addition reactions, the tunneling factors from harmonic and anharmonic gas-phase calculations are generally very similar. However, all tunneling factors for the addition reactions are small and, thus, less important for the total reaction. The trends for the tunneling factors for the addition reactions agree with the trends found for uracil, which underlines again that the anharmonic gas-phase calculations differ very little from the harmonic gas-phase calculations. The tunneling factors from the harmonic PCM calculations differ from those of the harmonic gas-phase calculations, showing that applying a PCM model of the solvent has an impact on the tunneling factor. However, when applying PCM and anharmonicity frequencies, the results are close to the harmonic PCM results, showing again that anharmonicity can be neglected in the calculation of the tunneling factors for the addition reactions.

When investigating the imaginary frequency for both addition and abstraction pathways, the tendencies are similar to what was observed for uracil. The imaginary frequencies from the harmonic and anharmonic gas-phase calculations are almost identical. This trend also applies to the solvent harmonic and anharmonic calculations, where their respective imaginary frequencies are comparable. This stresses again that for the imaginary frequencies the anharmonic inclusion has little impact when compared to harmonic imaginary frequencies. The comparison of anharmonic frequency with PCM and harmonic frequency with PCM underlines that anharmonic frequency can be neglected. The inclusion of solvent has an important impact on the imaginary frequencies, which can be seen by comparing the gas-phase harmonic and solvent harmonic calculations. For the imaginary frequencies, it is thus a fair assumption to use only harmonic frequencies; however, the solvent cannot be neglected.

**Figure 4:**
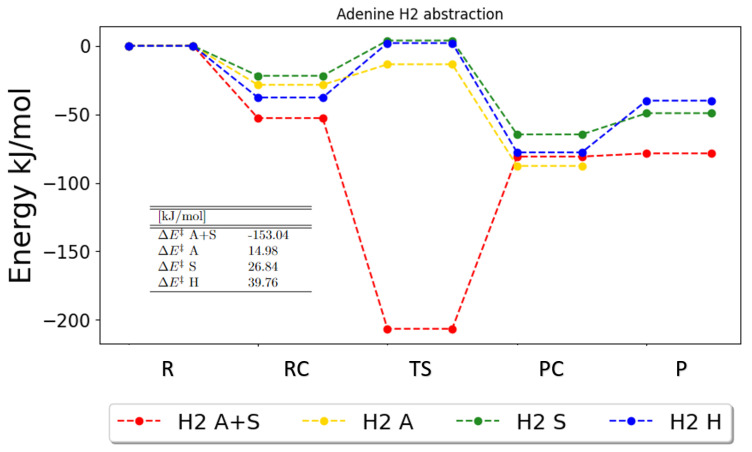
Zero-point vibrational corrected energies for the abstraction of H2 in adenine. A+S indicates anharmonic PCM calculation, A indicates anharmonic gas-phase calculations, S indicates harmonic PCM calculations and H indicates harmonic gas-phase calculations. R, RC, TS, PC and P denote the reactant, the reactant complex, the transition state, the product complex and the products, respectively.

Another contribution to the rate constants is the barrier height. PESs of the zero-point vibrational corrected energies for the different reaction pathways for adenine are therefore discussed in the following. The H2 abstraction reaction, shown in Figure 4, is a special case. For the PCM calculations with anharmonic frequencies (the H2 A+S red graph in Figure 4), the anharmonic zero-point vibrational correction to the energy is much smaller for the transition state than for the reactants, products and reactant and product complexes, which causes the zero-point corrected energy of the transition state to be lower than the corresponding energies of the reactant or product complexes. This implies that neither an Eckart tunneling factor nor a rate constant can be calculated with transition state theory, i.e., from Equation (4). Otherwise, the energetics from the anharmonic, the solvent and the anharmonic and solvent calculations seem to be relatively close for this reaction.

**Figure 5:**
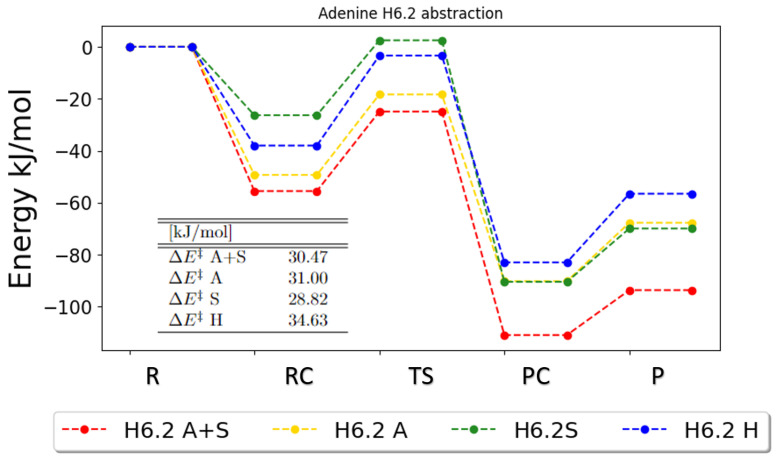
Zero-point vibrational corrected energies for the abstraction of H6.2 in adenine. A+S indicates anharmonic PCM calculation, A indicates anharmonic gas-phase calculations, S indicates harmonic PCM calculations and H indicates harmonic gas-phase calculations. R, RC, TS, PC and P denote the reactant, the reactant complex, the transition state, the product complex and the products, respectively.

For the H6.1 and H6.2 abstractions, the PESs are very similar. Figure 5 thus shows only the PES for the abstraction of H6.2. It can be observed that the anharmonic gas-phase and solvent energies, on one hand, are more similar to each other and the harmonic gas-phase and solvent energies, on the other hand, are more similar as well. This tendency differs from every other observed trend so far and implies that for these reactions the inclusion of anharmonic effects has an impact on the results. Although the harmonic solvent curve has the highest zero-point corrected energy for the transition state and the anharmonic solvent curve the lowest, the forward energy barriers are comparable for all models.

**Figure 6:**
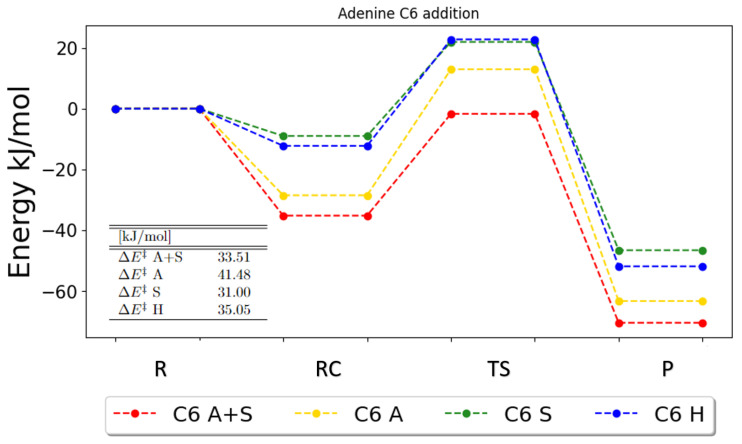
Zero-point vibrational corrected energies for the addition onto C6 in adenine. A+S indicates anharmonic PCM calculation, A indicates anharmonic gas-phase calculations, S indicates harmonic PCM calculations and H indicates harmonic gas-phase calculations. R, RC, TS, PC and P denote the reactant, the reactant complex, the transition state, the product complex and the products, respectively.

In general, for the reactions with adenine, the tendencies for the energetics are that harmonic PCM and harmonic gas phase are in better agreement with one another, and the energies of the anharmonic calculations seem to agree more with each other, i.e., a different pattern than what we saw for uracil. This is very clear for the additions at C4, C5, C6 and C8, where it seems that anharmonicity has a greater impact than the solvent, as illustrated for C6 in Figure 6.

The rate constants for the different reactions in adenine thus also differ from the trend observed for uracil. When applying the solvent model, we observe rate constants that are in most cases a bit smaller in magnitude than those from the harmonic gas-phase calculations, in agreement with what we observed for uracil. However, for some reactions, such as the C2 and C5 addition, the PCM rate constants are found to be a bit larger than the harmonic gas-phase calculated rate constants.

When including anharmonicity, the reaction rate constants do not seem to follow the same pattern as observed for uracil. For some pathways, i.e., the anharmonic PCM calculations for abstraction of H2, the energetics change; thus, the zero-point corrected energy barrier becomes negative, and neither the tunneling factor can be calculated using the Eckart approximation nor the rate constant can be calculated using transition state theory, as discussed above. For the anharmonic gas-phase reactions, we also observe an unexpected pattern where the rate constants are of a higher magnitude than in the harmonic gas-phase calculations. There does not seem to be a general trend as to how much larger the anharmonic gas-phase rate constants are than the harmonic gas-phase ones. However, for both abstraction of H6.1 and addition onto C2, they differ by about $10^{4}$ and $10^{3}$, respectively. In general, the anharmonic zero-point energy correction can tip the energies and thus completely change the rate constants, as the energy of the barrier enters in the exponential term in the expression for the rate constants (Equation (4)). A small change in the barrier can lead to a large change in the rate constant. This implies that the anharmonic zero-point corrections affect the reaction rate constants for adenine more than for uracil, leading to noticeably different results from the harmonic gas-phase results.

It is important to note, though, that the relative order of the rate constants between the different pathways is preserved. This means that application of the different models affects the rate constant, but the relative tendencies for rate constants remain the same and the same as found in gas phase and in the literature, as already discussed. The rate constants are found to be largest for anharmonic gas-phase calculations for the abstraction reactions, largest for the anharmonic PCM calculations for the addition reactions and smallest for harmonic PCM calculations for abstraction and addition, which agrees with what was found for uracil.

Based on these results, we conclude that it is valid to exclude the effects of anharmonicity to some extent, since their calculation is very expensive computer-time-wise, but that it is important to include a solvent model to model the most realistic scenario for the reaction between an OH radical and a nucleobase in an aqueous environment. In the rest of this project, the solvent effects have been included, since they generally have the largest impact on the different parameters discussed.

### 2.2. Rate Constants and Tunneling Factors for Abstraction Reactions

When drawing conclusions on the reactions between an OH radical and a nucleobase, the thermodynamic aspect should not stand alone, since the abstraction reactions are not in thermodynamic equilibrium. In the following, the rate constant of the individual pathways calculated with harmonic frequencies and the PCM solvation model will be discussed, as well as the Eckart tunneling factor and the total reaction rate.

Cytosine has four abstractable hydrogens, two from the amine and two directly from the ring, whereas thymine has three abstractable hydrogens in the methyl group and two from the ring and uracil has three abstractable hydrogens on the ring. For the pyrimidines, the total rate constants for abstractions are found to be $5.52\times 10^{-13}$, $1.02\times 10^{-15}$ and $1.09\times 10^{-16}$ cm^3^ molecules^−1^s^−1^ for thymine, cytosine and uracil, respectively. The difference in the rate constants between thymine and the other pyrimidines is due to a very favorable abstraction of any of the three hydrogens in the methyl group in thymine. The individual pathways and total rates for abstraction reactions can be found in Table 5. The overall structure of the transitions states is not changed from the gas-phase calculations performed by Milhøj and Sauer [14] and we therefore refer the reader to the figures of the transition states presented in their work.

For thymine, the abstraction of a methyl hydrogen, here illustrated by the abstraction of H7.1, is preferred as expected, and compared to the other abstractions within thymine, it is the dominant and most realistic pathway. For cytosine, all pathways seem reasonable, and for uracil, the abstraction of H6 seems favored. When comparing to the gas-phase results [14], there is a consistency in the favored trends; however, the rate constants are found to be smaller for the reactions including water as a solvent. For thymine and cytosine, the total rate constants are found to be two orders of magnitude smaller and for uracil, the total rate constant is found to be three orders of magnitude smaller than for the gas phase.

Adenine and guanine both have four abstractable hydrogens, whereas two of them are an amine group. For the purines, all abstractions seem plausible but that of H8. However, for adenine, the most preferred pathways are abstractions of the amine group (H6.1 or H6.2) and the similar abstractions would be expected to be the fastest for guanine; however, the results for guanine could not been obtained. The results for guanine were not reached due to problems finding the transition state using the Berny algorithm and due to problems optimizing the correct reactant complex, which prevented us from employing the QST2 method.

As seen for the pyrimidines, the comparison between the purine rate constants in solvent and the rate constants obtained in gas phase shows that the trends and favored pathways are similar. However, the total rate constant for adenine is found to be one order of magnitude smaller in solvent than in gas phase. In general, we can conclude that the reaction rates become a bit smaller when applying a solvent model in the calculations.

### 2.3. Rate Constants and Tunneling Factors for Addition Reactions

For the addition reaction of OH onto a carbon atom in the nucleobases, the individual and total rate constants are given in Table 6. Again, we refer the reader to the work of Milhøj and Sauer [14] for figures of the transition states. For the purines, addition onto C6 seems unfavorable, whereas for adenine, the addition onto C8 is the biggest contribution to the total rate constant. The individual pathways are found to be several orders of magnitude smaller for the addition reactions compared to the abstraction reactions, except for the addition onto C8, which generally is the preferred individual pathway for adenine.

The obtained rates in solvent can be compared to the gas-phase results [14]. The comparison shows agreement for the tendencies for each individual rate, which also agrees with the literature discussed in the Introduction. For the purines, the plausible addition reactions are found to be C8; however, every addition apart from C6 can happen, but the preferred addition would be the one that breaks the aromaticity the least. The rates obtained in solvent are also found to be a bit smaller for the addition reactions. For adenine, the total rate constant is two orders of magnitude smaller in solvent phase than in gas phase.

For the pyrimidines, the C5 results could not be obtained, since all our attempts to find a transition state for addition at C5 ended in a transition state for addition to C6. It is, however, expected that addition onto the C6–C5 double bound is the preferred pathway. The individual rates for all of the pyrimidines give rise to this, since the rate constant for addition onto C6 is clearly the biggest contribution to the overall rate constant. This agrees with the trends from gas-phase calculations. For all pyrimidines, addition onto C2 or C4 is unlikely.

All of this suggests that additions might give the most dominant contribution to the overall reaction rate. However, based on these results, the abstraction reactions also play an important role in the overall reaction in radiation damage of all nucleobases.

#### The Total Rate Constants

The total rate constant for each nucleobase was calculated using Equation (5). The total rate constants are shown in Table 7, where the experimental values from Scholes [41] are given as well. In general, the total rate constants are about two orders of magnitude smaller than the experimental values. For the three pyrimidines, this might be due to the lack of results for the addition to C5. Based on the individual reaction rates, it should be noted that even if the abstraction reactions are not necessarily the most important path in the radiation damage for all nucleobases, they do play an important role in order to obtain the general picture and account for every possible path.

### 2.4. Gibbs Energies

The Gibbs energies, free energies of activation $\Delta G^{‡}$ and free energies of reaction $\Delta_{r}G$, are calculated as indicators of kinetic and thermodynamic stability and pathway preferences as follows:

(1) $$\Delta G^{‡}=\Delta G_{TS}-\left(\Delta G_{R}+\Delta G_{OH}\right)$$

(2) $$\Delta_{r}G=\left(\Delta G_{P}+\Delta G_{H_{2}O}\right)-\left(\Delta G_{R}+\Delta G_{OH}\right)$$

(3) $$\Delta_{r}G=\Delta G_{P}-\left(\Delta G_{R}+\Delta G_{OH}\right)$$

The free energy of activation, (1), predicts the relative sizes of the rate constant, whereas the free energies of reaction, (2) and (3), predict the thermodynamic stability of the reactant and products. Equation (2) is used for the abstraction reactions, where the products are a radical and water. Equation (3) is used for the addition reaction. However, since the products of the abstraction reactions are highly reactive radicals, we assume that thermodynamic equilibrium is not upheld, since the radical is highly unstable and is expected to react further. As discussed, this makes it difficult to conclude anything based on the thermodynamic stabilities alone. The Gibbs free energies are plotted in the following for each individual reaction pathway in order to obtain thermodynamic guidance on favored reactions.

#### 2.4.1. Gibbs Energies for Abstraction Reactions

**Figure 7:**
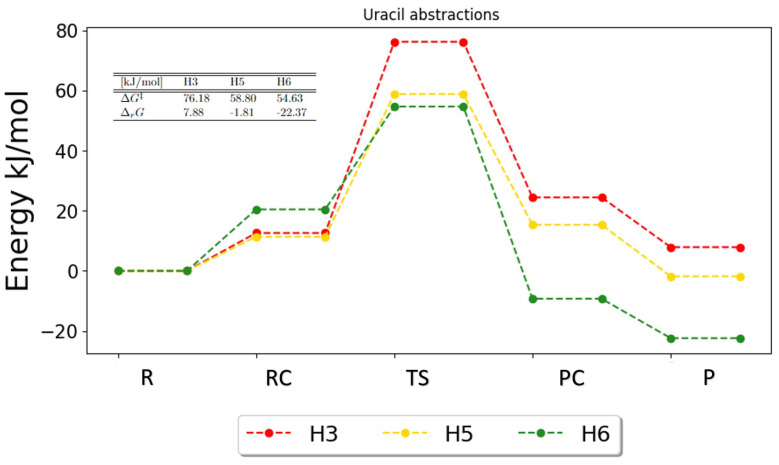
Potential energy surface for the possible abstractions in uracil. The free energies of activation and reaction are given in the table. R, RC, TS, PC and P denote the reactant, the reactant complex, the transition state, the product complex and the products, respectively.

**Figure 8:**
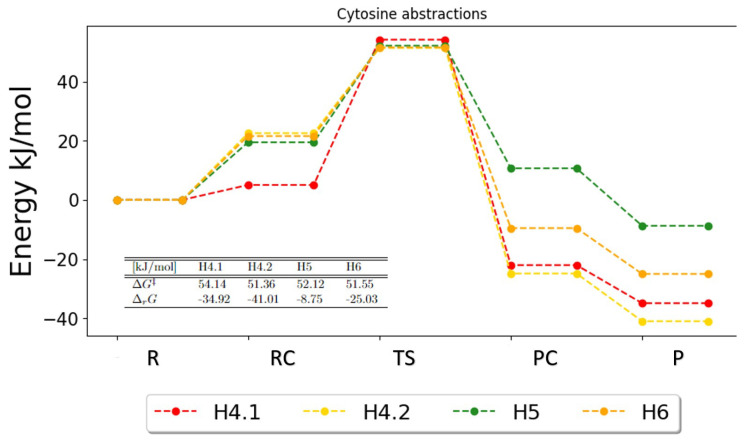
Potential energy surface for the possible abstractions in cytosine. The free energies of activation and reaction are given in the table. R, RC, TS, PC and P denote the reactant, the reactant complex, the transition state, the product complex and the products, respectively.

**Figure 9:**
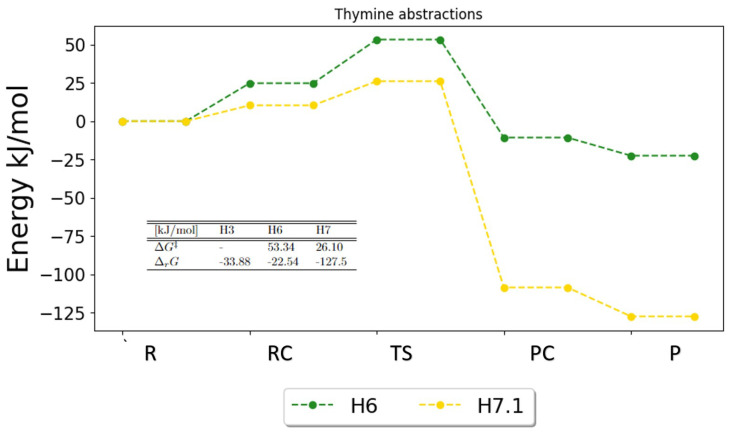
Potential energy surface for the possible abstractions in thymine. The free energies of activation and reaction are given in the table. The full set of results for H3 has not been obtained.

The potential energy surfaces (PESs) for the hydrogen abstraction reaction for the pyrimidines are show in Figure 7, Figure 8 and Figure 9. These figures indicate preferred pathways and agree with the reaction rates using the transition state theory and Eckart tunneling factor.

For uracil, the PES in Figure 7 is obtained for each abstraction reaction. The H3 abstraction is least favorable due to multiple reasons. The free energy of activation is much higher for H3 than for any of the other abstractions. This indicates a large barrier, as the PES also shows. Since H3 has the largest free energy of activation, based on Gibbs energetics only, we would expect H3 to have the smallest rate constant. The abstraction of H6 has the smallest energy barrier, which indicates that this reaction is most likely to occur. The free energies of activation indicate that H6 must be more favorable, since the difference between the reactant and the product is largest. The PES also shows that the product for H6 has the lowest Gibbs energy and is thereby most thermodynamic stable, whereas the abstraction of H3 is the least thermodynamic stable, since the reaction energy is positive, which means that the product is higher in energy than the reactant.

The potential energy surfaces for abstractions in cytosine are shown in Figure 8. Based on these PESs, every reaction is likely to occur, which agrees with the previous conclusions from the analysis of the reaction rate constants. The free energies of activation for H4.2, H5 and H6 are all very close (51.36 kJ/mol, 52.12 kJ/mol and 51.55 kJ/mol, respectively), whereas the activation energy for H4.1 is slightly higher (54.14). The activation energies for every pathway lie in a close interval and based on this, every reaction still seems plausible. The biggest difference is found in the reaction energies, where the abstractions of H4.1 and H4.2 are lowest (−34.92 kJ/mol, −41.01 kJ/mol) and that of H6 is highest (−8.75 kJ/mol). It is unarguable that every reaction might occur, since the product for each abstraction leads to a lower energy, which is preferable. Based on the Gibbs activation and reaction energies, it seems possible that every reaction will contribute to the overall reactions.

In Figure 9, the PES for thymine is shown. The PES for thymine shows a one-sided picture, that is, one abstraction reaction happens with great probability. Even though no results were obtained for the H3 abstraction, it is clear from the PES that the H7.1 abstraction is favored and dominant. According to the already-discussed literature, the H7.1 abstraction plays the most important role, and it is expected that the H3 abstraction is not likely to change this picture. Both the free activation energy (26.10 kJ/mol) and the free reaction energy (−127.55 kJ/mol) for the H7.1 abstraction are dominant. The free energies of reaction were calculated for every reaction: H3 (−33.88 kJ/mol), H6 (−22.54 kJ/mol) and H7.1 (−127.55 kJ/mol). H7.1 has a significantly much lower free reaction energy and the lowest free energy of activation; thus, this reaction is expected to be the single most important reaction for thymine abstractions.

**Figure 10:**
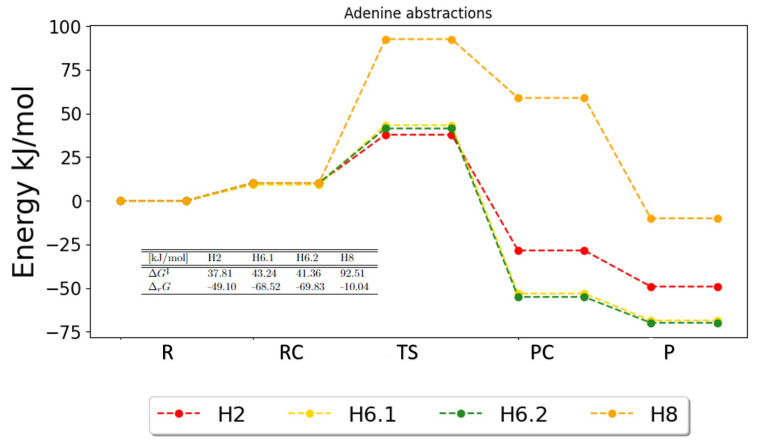
Potential energy surface for the possible abstractions in adenine. The free energies of activation and reaction are given in the table. R, RC, TS, PC and P denote the reactant, the reactant complex, the transition state, the product complex and the products, respectively.

For the purines, only the PES for adenine is shown in Figure 10. The PES for adenine, Figure 10, shows the expected similarities between H6.1 and H6.2. They have similar energy barriers, with activation energies of 43.24 kJ/mol and 41.36 kJ/mol, respectively, showing a slight preference for H6.1 based on the barriers. The lowest activation energy is obtained for H2 (37.81 kJ/mol). For the Gibbs free reaction energies, H6.1 and H6.2 are still comparable, with energies of −68.52 kJ/mol and −69.83 kJ/mol, respectively, which show a slight preference for H6.2. The reaction energy for H2 is −49.10 kJ/mol, which is smaller than for H6.1 and H6.2, so even if H2 obtained the lowest barrier, this reaction is not expected to be the most preferred due to the reaction energy. The abstraction of H8 is very unlikely to happen, since the energy from reactant to reactant complex is extremely high and, furthermore, there is a very small reaction energy (−10.04 kJ/mol) and therefore, little gain for the reaction. Based on the PES, abstraction of the amine H6.1 and H6.2 are most likely to occur and abstraction of H2 is plausible as well. Abstraction of H8 seems very unlikely. This agrees with what was found for the reaction rates.

#### 2.4.2. Gibbs Energies Addition Reactions

**Figure 11:**
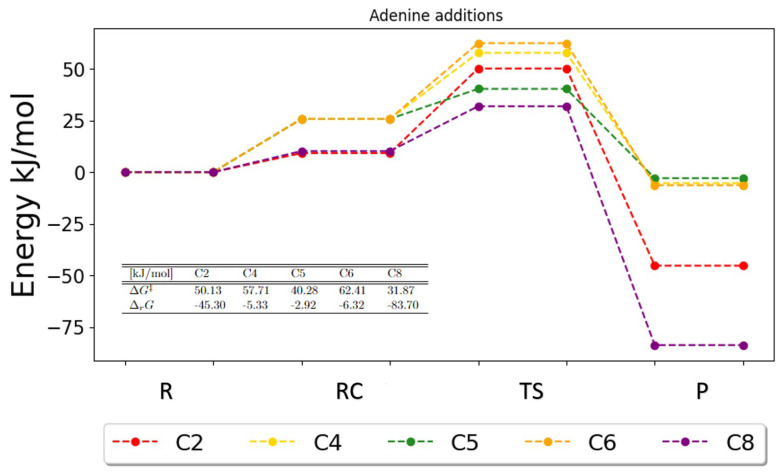
Potential energy surface for the possible addition reaction in adenine. The free energies of activation and reaction are given in the table. R, RC, TS and P denote the reactant, the reactant complex, the transition state and the products, respectively.

**Figure 12:**
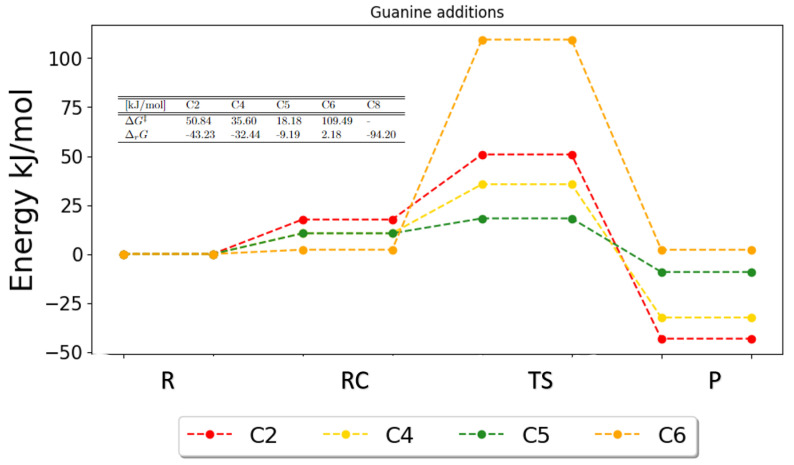
Potential energy surface for the possible addition reaction in guanine. The free energies of activation and reaction are given in the table. The full set of results for C8 has not been obtained. R, RC, TS and P denote the reactant, the reactant complex, the transition state and the products, respectively.

For the addition reactions, figures showing the Gibbs free energies were obtained as well. For the purines, the potential energy surfaces are plotted for the addition reactions in Figure 11 and Figure 12.

For adenine, Figure 11, it is clear that addition to C8 is the most favored. This is due to a low Gibbs free activation energy (31.87 kJ/mol) and a large Gibbs free reaction energy (−83.70 kJ/mol). Since these two energies for C8 are the lowest and largest, respectively, compared to any other addition for adenine, the addition of the OH radical onto carbon C8 must be the preferred addition. The C6 addition appears to be the least favored based on the high activation energy (62.41 kJ/mol) and the very small free reaction energy (−6.32 kJ/mol), indicating that there is very little energetic gain for the reaction to occur, i.e., it will likely not happen. A little energetic gain is also observed for C4 and C5 with a slight preference for C4. For the C2, C4 and C5 additions, the activation energies are 50.13 kJ/mol, 57.71 kJ/mol and 40.28 kJ/mol, respectively, which favors C5 slightly. Since the energy barrier is in the exponent in the reaction rate, a small difference in energy barriers has a huge impact on the rate constant. Thus, even though the free energy of reaction is significantly larger for C2 (−45.30 kJ/mol), the C5 reaction is assumed to occur more frequently due to the smaller barrier. The results based on the Gibbs energetics seem to be in agreement with the results obtained for the reaction rate calculations using transition state theory with zero-point corrected energies.

The PES for the addition reactions to guanine can be viewed in Figure 12. Results for the transition state of the addition onto C8 could not be obtained; thus, the PES is not shown. The free energy of reaction was obtained for C8, and this alone is a strong indicator that this reaction would be preferred based on the reaction energy (−94.20 kJ/mol), which is the largest of all addition reactions. This implies that there is a large energetic gain for this reaction to occur. Based on the Gibbs energies, there undoubtedly is a large activation barrier for C6 (109.49 kJ/mol), which indicates a low reaction rate and a small possibility for this reaction to occur. Moreover, there is no energetic gain for the C6 addition to occur, since the free energy of reaction is even positive (2.18 kJ/mol). The free energies of activation for C2, C4 and C5 are found to be 50.84 kJ/mol, 35.60 kJ/mol and 18.17 kJ/mol, respectively. This shows a preference for the C5 addition. However, their free energies of reaction are found to be −43.23 kJ/mol, −32.44 kJ/mol and −9.19 kJ/mol, respectively, and based on this alone, the largest energetic gain is found for C2. But, as already discussed, the free energies of activation and, thereby, the energy barriers play the most dominant role for the reaction rate, which is why we would expect C5 to be the most favorable of these three additions and C2 to be the least favorable. This agrees with the results obtained for the reaction rates.

**Figure 13:**
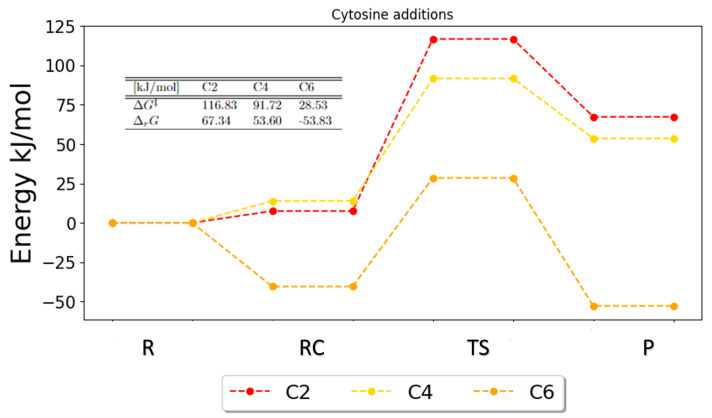
Potential energy surface for the possible addition reaction in cytosine. The free energies of activation and reaction are given in the table. The full set of results for C5 has not been obtained. R, RC, TS and P denote the reactant, the reactant complex, the transition state, the product and the products, respectively.

Figure 13 shows the PES for addition reactions in cytosine; however, the tendencies are similar to the tendencies for the other pyrimidines. It shows clearly that C6 is the preferred pathway based on both the free energies of activation and reaction. The PES for C5 is not shown, as it was not possible to obtain a transition state for this reaction. It also shows how C2 and C4 have larger barriers and no energetic gain; this indicates that these reactions would likely not occur.

From this, we can conclude that the Gibbs energetics support the conclusions obtained from the calculated reaction rates. The preferences for individual pathways when investigating Gibbs energies agree with the preferences for the individual pathways when investigating the reaction rates calculated with transition state theory from zero-point vibrational corrected energies and Eckart tunneling factors. Based on the Gibbs energies, it can also be stressed that the height of the barrier plays a more important role compared to the energetic gain that can be obtained through the reaction. This makes great sense, since the activation barrier is in the exponent for calculating the rates, and a small change in energy difference has a huge impact on the rate constant.

## 3. Materials and Methods

### 3.1. Theory

At the high-pressure limit, the reaction rate converges towards the principles of the transition state theory (TST) [42]. TST describes the trajectory of a reacting particle as it moves from one energy minimum to another by traversing a potential energy barrier that is a saddle point. At this saddle point, the structure is often referred to as an activated complex or the transition state. The height of the potential energy barrier is determined by the activation energy, which is represented by the difference in energy between the zero-point vibrational corrected energy of the reactant and that of the transition state.

TST is based on two primary assumptions. First, it assumes that thermodynamic equilibrium is upheld throughout the reaction, ensuring that the Boltzmann distribution is suitable for describing the particle distribution. The second assumption states that once the moving particle has crossed the potential energy barrier at the saddle point, it proceeds to the energy minimum of the product well without the possibility of recrossing. This assumption is valid for gas-phase reactions, where strain does not significantly impact the reaction. In reactions involving solvated reactants, strain and the possibility for collisions with solvent molecules introduce a breakdown in this assumption. In solvated reactions, the occurrence of recrossings can no longer be neglected. Transition state theory does account for solvent effects to some extent, considering both stabilization and destabilization. The solvent can either stabilize or destabilize reactants, products or transition states through intermolecular forces, influencing the favorability of the reaction. If the solvent stabilizes the transition state while destabilizing the reactants, it results in a reduction in the activation energy and, consequently, affects the reaction rate.

In order to obtain the most realistic reaction rates, recrossing effects should be accounted for by applying a correction factor such as Kramers’ correction or the Grote–Hynes correction factor [43,44]. However, the solvent effects in regards to recrossings are beyond the scope of this project.

The reaction rate constants for the addition and abstraction reactions are calculated with transition state theory, where the rate can be expressed as

(4) $$k=\sigma \kappa \left(T\right)\frac{k_{b}T}{h}\frac{Q_{TS}}{Q_{R}Q_{OH}}\exp\left(-\frac{\Delta E^{‡}}{k_{b}T}\right)$$

where σ denotes the symmetry factor, which is the number of identical reaction paths, κ denotes the tunneling factor, $\Delta E^{‡}$ denotes the activation barrier, which is the energy difference between the transition states’ zero-point vibrational corrected energies and the reactants’ zero-point vibrational energies. *Q* denotes the total partition function, using the non-imaginary frequencies. A planar transition state gives rise to a symmetry factor value of 1 whereas a non-planar transition state gives rise to axial chirality. The total rate constant can be calculated as the sum of individual rate constants:

(5) $$k_{total}=\Sigma_{i}k_{i}$$

These results can be directly compared to experimental values.

The quantum mechanical phenomenon tunneling describes a particle’s ability to penetrate through an energy barrier with higher energy than the kinetic energy of the particle. If the tunneling factor is 1, it means that no tunneling is observed. For reactions where tunneling is observed, tunneling factors, κ(T), can be calculated through different approaches. In this study, the tunneling factor was calculated as the Eckart tunneling factor, which can be calculated by an integral of the transmission probability [17].

(6) $$\kappa \left(T\right)=\frac{\exp(\frac{-\Delta E_{fwd}}{k_{b}T})}{k_{b}T}\int_{0}^{\infty }P_{QM}\left(E\right)\exp\left(-\frac{E}{k_{b}T}\right)\;dE$$

(7) $$P_{QM}\left(E\right)=1-\left(\frac{\cosh(\alpha -\beta )+\cosh(\delta )}{\cosh(\alpha +\beta )+\cosh(\delta )}\right).$$

$P_{QM}\left(E\right)$ is the probability of quantum mechanical tunneling, *E* denotes the energy, and $\Delta E_{fwd}$ is the forward energy barrier. The Eckart tunneling factor includes both the forward zero-point vibrational corrected energy barrier and the backward zero-point vibrational corrected energy barrier, and is therefore a very exact tunneling factor. α, β and δ are related to the backward and the forward zero-point corrected energy barriers, as discussed in detail in previous work [20]. In this project, the Eckart factor was calculated by numerical integration. It is noted that the Eckart factor is temperature dependent; however, an increase in temperature does not necessarily mean an increase in reaction rate. This is because a higher temperature allows more molecules to obtain enough energy to overcome a potential energy barrier without tunneling through it. It is arguable that the Eckart tunneling factor overestimates the tunneling, since it gives rise to too-narrow barriers. A tunneling factor such as small curvature tunneling would be a better tunneling approximation [45]. This, however, is beyond the scope of this research.

### 3.2. Computational Methods

Previous research within our group has investigated different computational approaches in order to obtain the best results for reaction rate calculations for the reaction between a nucleobase and a hydroxyl radical [13]. It was found that DFT calculations employing the ωB97X-D exchange-correlation functional [15] with the 6-311++G(2df,2pd) basis set [16] and the Eckart tunneling correction factor [17] give the most precise results. It should be noted that the basis set employed here is significantly larger than those employed in previous calculations by other groups.

All calculations were carried out with the Gaussian16 program suite [46]. Geometry optimizations were first conducted, followed by frequency calculations. The implicit solvent model PCM in its integral equation formalism (IEF-PCM) [18,19] was employed for the aqueous environment in every step of the calculations. Previous work from our group has proven the PCM model to be a reasonable solvent model for these calculations, since it showed good agreement with experimental results for thymine [21]. The article concluded that the PCM solvent model and the Eckart tunneling factors gave overall results in best agreement with experimental data. When applying the PCM solvent model, the calculations lack the inclusion of some solvent effects such as explicit hydrogen bonds. To expound for such solvent effects, explicit water molecules should be included in the calculations. In another study on adenine, including an explicit water molecule in addition to PCM gave results in good agreement with experimental values for adenine [20]. However, as discussed in [21], this leads to a double count of electrostatic effects, impacting the results. In order to have the best possible basis of comparison for reaction constants in this project, the same solvation model (PCM) is used for all calculations.

The transition state (TS) structures were optimized using the standard Berny algorithm in Gaussian16 [47,48] and controlled by a frequency inspection and a visual analysis that assures only one imaginary frequency for the TS. The reactions were controlled by IRC calculations from the TS, the following associated product complexes’ and reaction complexes’ geometries were optimized to a minimum and the frequencies were calculated [49]. However, not all results could be checked with IRC, due to difficulties finding the correct transition states for some of the reactions. These results are marked with [a] in the following tables.

Not every reaction rate constant was obtained, since we experienced difficulties finding the correct transition states. For the pyrimidines, the addition onto C5 was difficult to obtain by Berny optimization. The QST2 method [50] was tried for uracil C5. However, for thymine and cytosine, there were problems optimizing the correct reactant complex. For guanine abstractions, every transition state proved difficult to obtain. It has been highlighted throughout the following whenever a problem with finding the correct transition state was met.

The calculation of the anharmonic vibrational frequencies was carried out with the FREQ = ANHARM keyword in the Gaussian16 program suite.

In order to determine the importance of anharmonicity and solvent effects on the reaction between an OH radical and a nucleobase, three different calculations for reaction pathways in adenine and uracil were obtained, including anharmonic corrections to the frequencies in a gas-phase calculation with harmonic frequencies and the PCM solvent model and, finally, a combination of an anharmonic frequency calculation with the PCM solvent model. For the rest of the nucleobases, the pathways were only investigated employing the PCM solvent model.

## 4. Conclusions

Thermodynamic and kinetic considerations were examined to account for the importance of anharmonicity and solvent effects for reactions between an OH radical and a nucleobase. Generally, the investigations suggest that the solvent has a more pronounced impact on the Eckart tunneling factor, the imaginary frequencies and the zero-point vibrational energy compared to the inclusion of anharmonicity. It appears that the addition of a solvent via the PCM model reduces the rate constant by several orders of magnitude. The imaginary frequency and tunneling factors seem unaffected by the application of anharmonicity; however, it looks like the anharmonic correction can affect the potential energy surface, which in some cases makes it impossible to apply transition state theory for the calculation of the rate constants. Overall, it is apparent that the presence of solvent has a crucial influence on several parameters, while the effect of anharmonicity has less influence and impacts fewer parameters. To determine whether one can omit one of these corrections, one must determine how much influence the anharmonic aspect has on the rate constant relative to the computational expense and time required. In this project, it was concluded that the contribution from anharmonicity does not impact the overall picture, and the inclusion has, therefore, been omitted when calculating the total rate constant for each of the five nucleobases.

Most of the reaction rates were calculated with PCM for every individual pathway in order to determine the importance of the different paths. The calculated Gibbs free energies of activation and rate constants lead to the same conclusions. For the purines, the addition to C8 is the predominant addition reaction and gives the biggest contribution to the overall reaction rate. For the pyrimidines, the C6 addition is most likely to occur; however, we expect it to be a close competition between addition onto C6 or C5.

The most important abstraction is the abstraction of a methyl hydrogen of thymine. This abstraction competes directly with the addition reactions, which indicates that even though the addition reaction is dominant, the abstraction reaction cannot be disregarded. For cytosine, all abstractions are plausible, and for uracil, H6 is found to be the most likely. For adenine, the amine abstractions are most likely to occur, and we would anticipate guanine to react alike.

From this, we can conclude that applying a solvent model in the simulations of the reactions affects the magnitude of the total and individual reaction rates. However, the relative order of the rates remains unchanged. Applying anharmonicity corrections to the vibrational frequencies can tip the energy balance and create scenarios where transition state theory is no longer applicable for the calculation of rate constants. However, in the general picture, anharmonicity impacts fewer parameters compared to the solvent.

## Figures and Tables

**Table 1:** Rate constant for uracil H abstractions calculated at ωB97X-D/6-311++G(2df,2pd) level. The rate constants are given in units of cm^3^ molecules^−1^s^−1^ and at ambient conditions: T=298 K and p=1 atm. In parentheses, the Eckart tunneling factor and the imaginary frequency [cm^−1^] are given.

Uracil	H3	H5	H6
Harmonic gas phase [14] ^[a]^	4.43 $\times \;10^{-19}$ (17.42, 1614.63)	2.05 $\times \;10^{-16}$ (18.41, 1684.03)	3.01 $\times \;10^{-15}$ (23.14, 1567.34)
Anharmonic gas phase	1.41 $\times \;10^{-18}$ (17.81, 1602.06)	5.02 $\times \;10^{-16}$ (19.81, 1678.88)	3.47 $\times \;10^{-15}$ (23.12, 1572.15)
Harmonic with PCM	3.30 $\times \;10^{-20}$ (53.55, 1812.04)	9.33 $\times \;10^{-18}$ (27.29, 1713.91)	1.09 $\times \;10^{-16}$ (29.75, 1685.69) ^[b]^
Anharmonic with PCM	1.76 $\times \;10^{-20}$ (64.47, 1811.21)	4.27 $\times \;10^{-17}$ (32.75, 1713.14)	3.74 $\times \;10^{-17}$ (33.19, 1684.71) ^[b]^

^[a]^ The Eckart tunneling factors and thus, the rate constants presented here differ from the ones in this article, as the Eckart tunneling factors had to be recalculated here due to an error in the previous calculations. ^[b]^ indicates that the calculations have not been checked with IRC calculations.

**Table 2:** Rate constant for uracil additions calculated at ωB97X-D/6-311++G(2df,2pd) level. The rate constants are given in units of cm^3^ molecules^−1^s^−1^ and at ambient conditions: T=298 K and p=1 atm. In parentheses, the Eckart tunneling factor and the imaginary frequency [cm^−1^] are given.

Uracil	C2	C4	C5	C6
Harmonic gas phase [14] ^[a]^	8.12 $\times \;10^{-29}$	1.12 $\times \;10^{-23}$	2.38 $\times \;10^{-12}$	2.44 $\times \;10^{-12}$
	(1.64, 679.03)	(1.45, 592.03)	(1.05, 247.27)	(1.05, 246.96)
Anharmonic gas phase	1.47448 $\times \;10^{-28}$	1.30 $\times \;10^{-23}$	1.29 $\times \;10^{-12}$	4.08 $\times \;10^{-12}$
	(1.63, 676.47) ^[b]^	(1.43, 580,94)	(1.07, 267.53)	(1.05, 233.34)
Harmonic with PCM	5.18 $\times \;10^{-28}$	7.36 $\times \;10^{-26}$	—– ^[c]^	7.58 $\times \;10^{-14}$
	(1.83, 747.12)	(1.41, 572.37)		(1.04, 197.48)
Anharmonic with PCM	2.45 $\times \;10^{-28}$	5.91E $\times \;10^{-26}$	—– ^[c]^	7.52 $\times \;10^{-13}$
	(1.83, 747.59)	(1.41, 572.41)		(1.04, 198.73) ^[b]^

^[a]^ The Eckart tunneling factors and thus, the rate constants presented here differ from the ones in this article, as the Eckart tunneling factors had to be recalculated here due to an error in the previous calculations. ^[b]^ indicates that the calculations have not been checked with IRC calculations. ^[c]^ could not be found, since the OH radical jumps to C6.

**Table 3:** Rate constant for adenine H abstractions calculated at ωB97X-D/6-311++G(2df,2pd) level. The rate constants are given in units of cm^3^ molecules^−1^s^−1^ and at ambient conditions: T=298 K and p=1 atm and in parentheses are given the Eckart tunneling factor and the imaginary frequencies.

Adenine	H2	H6.1	H6.2	H8
Harmonic gas phase [14] ^[a]^	1.54 $\times \;10^{-14}$	1.53 $\times \;10^{-13}$	8.36 $\times \;10^{-13}$	2.54 $\times \;10^{-24}$
	(4.01, 1106.18)	(35.69, 1765.47)	(66.74, 1962.84)	(2.26 $\times \;10^{9}$, 2987.07)
Anharmonic gas phase	8.65 $\times \;10^{-12}$	3.17 $\times \;10^{-9}$	1.83 $\times \;10^{-10}$	7.56 $\times \;10^{-22}$
	(3.02, 1090.13) ^[b]^	(28.88, 1770.59) ^[b]^	(48.16, 1939.95) ^[b]^	(1.19 $\times \;10^{9}$, 2979) ^[b]^
Harmonic with PCM	1.43 $\times \;10^{-15}$	1.0718 $\times \;10^{-14}$	9.74 $\times \;10^{-14}$	1.44 $\times \;10^{-26}$
	(1.76, 735.44)	(29.54, 1774.31)	(125.7, 2391.83)	(1.17, 395.27) ^[b]^
Anharmonic with PCM	N/A ^[c]^	9.70 $\times \;10^{-11}$	1.24 $\times \;10^{-11}$	1.86 $\times \;10^{-17}$
		(32.48, 1774.43) ^[b]^	(156.7, 2391.55) ^[b]^	(—, 395.85) ^[b]^

^[a]^ The Eckart tunneling factors and thus, the rate constants presented here differ from the ones in this article, as the Eckart tunneling factors had to be recalculated here due to an error in the previous calculations. ^[b]^ indicates that the calculations have not been checked with IRC calculations. ^[c]^ could not be obtained due to a breakdown of TST.

**Table 4:** Rate constant for adenine additions calculated at ωB97X-D/6-311++G(2df,2pd) level. The rate constants are given in units of cm^3^ molecules^−1^s^−1^ and at ambient conditions: T=298 K and p=1 atm and in parentheses are given the Eckart tunneling factor and the imaginary frequencies.

Adenine	C2	C4	C5	C6	C8
Harmonic gas phase [14] ^[a]^	9.51 $\times \;10^{-18}$	2.60 $\times \;10^{-18}$	5.52 $\times \;10^{-16}$	2.74 $\times \;10^{-19}$	1.10 $\times \;10^{-12}$
	(1.33, 521.53)	(1.28, 484.88)	(1.22, 434.51)	(1.33, 522.92)	(1.03, 216.34)
Anharmonic gas phase	3.18 $\times \;10^{-15}$	1.04 $\times \;10^{-15}$	8.14 $\times \;10^{-14}$	1.43 $\times \;10^{-17}$	7.89 $\times \;10^{-11}$
	(1.32, 515.97) ^[b]^	(1.28, 484.99) ^[b]^	(1.21, 430.49) ^[b]^	(1.32, 518.40) ^[b]^	(1.05, 216.06) ^[b]^
Harmonic with PCM	2.84 $\times \;10^{-17}$	1.26 $\times \;10^{-18}$	1.41 $\times \;10^{-15}$	1.98 $\times \;10^{-19}$	3.67 $\times \;10^{-14}$
	(1.26, 476.65)	(1.19, 405.12)	(1.17, 339.94)	(1.24, 453.40)	(1.03, 173.77)
Anharmonic with PCM	3.06 $\times \;10^{-12}$	1.29 $\times \;10^{-14}$	4.95 $\times \;10^{-11}$	2.69 $\times \;10^{-15}$	8.52 $\times \;10^{-10}$
	(1.26, 478.13) ^[b]^	(1.19, 405.34) ^[b]^	(1.13, 340.15) ^[b]^	(1.24, 454.13) ^[b]^	(1.03, 173.06)

^[a]^ The Eckart tunneling factors and thus, the rate constants presented here differ from the ones in this article, as the Eckart tunneling factors had to be recalculated here due to an error in the previous calculations. ^[b]^ indicates that the calculations have not been checked with IRC calculations.

**Table 5:** Rate constant for H abstraction reactions calculated at ωB97X-D/6-311++G(2df,2pd) level with the IEF-PCM solvent model. The rate constants are given in units of cm^3^ molecules^−1^s^−1^ and at ambient conditions: T=298 K and p=1 atm and in parentheses is given the Eckart tunneling factor.

**Adenine**	**H2**	**H6.1**	**H6.2**	**H8** ^[a]^	**Total**
	1.43 $\times \;10^{-15}$ (1.76)	1.07 $\times \;10^{-14}$ (29.54)	9.74 $\times \;10^{-14}$ (125.7)	9.94 $\times \;10^{-25}$ (1.17)	1.10 $\times \;10^{-13}$
**Thymine**	**H3**	**H6**	**H7.1**		**Total**
	—– ^[b]^	8.20 $\times \;10^{-17}$ (13.3)	5.52 $\times \;10^{-13}$ (1.51)		5.52 $\times \;10^{-13}$
**Cytosine**	**H4.1**	**H4.2**	**H5**	**H6**	**Total**
	4.54 $\times \;10^{-16}$ (102.4)	2.73 $\times \;10^{-16}$ (19.85)	1.29 $\times \;10^{-16}$ (12.77)	1.61 $\times \;10^{-16}$ (12.62)	1.02 $\times \;10^{-15}$
**Uracil**	**H3**	**H5**	**H6** ^[a]^		**Total**
	3.30 $\times \;10^{-20}$ (53.55)	9.33 $\times \;10^{-18}$ (27.29)	1.09^−16^ (29.73)		1.18 $\times \;10^{-16}$

^[a]^ indicates that the calculations have not been checked with IRC calculations. ^[b]^ could not be found.

**Table 6:** Rate constant for addition reactions calculated at ωB97X-D/6-311++G(2df,2pd) level with the IEF-PCM solvent model. The rate constants are given in units of cm^3^ molecules^−1^s^−1^ and at ambient conditions: T=298 K and p=1 atm and in parentheses is given the Eckart tunneling factor.

**Adenine**	**C2**	**C4**	**C5**	**C6**	**C8**	**Total**
	2.84 $\times \;10^{-17}$ (1.26)	1.26 $\times \;10^{-18}$ (1.19)	1.41 $\times \;10^{-15}$ (1.17)	1.98 $\times \;10^{-19}$ (1.24)	3.67 $\times \;10^{-14}$ (1.03)	3.81 $\times \;10^{-14}$
**Guanine**	**C2**	**C4**	**C5**	**C6**	**C8**	**Total**
	2.25 $\times \;10^{-17}$ (1.33)	9.11 $\times \;10^{-15}$ (1.15)	1.02 $\times \;10^{-11}$ (1.14)	1.29 $\times \;10^{-27}$ (1.43)	— ^[a]^	1.02 $\times \;10^{-11}$
**Thymine**	**C2**	**C4**	**C5**	**C6**		**Total**
	1.09 $\times \;10^{-27}$ (1.44)	3.10 $\times \;10^{-26}$ (1.42)	— ^[b]^	1.06 $\times \;10^{-14}$ (—) ^[c]^		1.06 $\times \;10^{-14}$
**Cytosine**	**C2**	**C4**	**C5**	**C6**		**Total**
	2.11 $\times \;10^{-29}$ (1.80)	1.62 $\times \;10^{-24}$ (1.39)	— ^[b]^	1.46 $\times \;10^{-13}$ (1.06)		1.46 $\times \;10^{-13}$
**Uracil**	**C2**	**C4**	**C5**	**C6**		**Total**
	5.18 $\times \;10^{-28}$ (1.83)	7.36 $\times \;10^{-26}$ (1.41)	— ^[b]^	7.58 $\times \;10^{-14}$ (1.04)		7.58 $\times \;10^{-14}$

^[a]^ could not be found. ^[b]^ could not be found, since the radical jumps to C6. ^[c]^ indicates that the Eckart tunneling factor has not yet been calculated.

**Table 7:** The total rate constant calculated at ωB97X-D/6-311++G(2df,2pd) level with the IEF-PCM solvent model and in gas phase [14]. The rate constants are given in units of cm^3^ molecules^−1^s^−1^ and at ambient conditions: T=298 K and p=1 atm.

Nucleobase	Total in Solvent	Total in Gas Phase [14]	Experimental [41]
Adenine	1.48 $\times \;10^{-13}$	2.17 $\times \;10^{-12}$	0.71–1.15 $\times \;10^{-11}$
Guanine	1.02 $\times \;10^{-11}$	5.64 $\times \;10^{-11}$	1.53 $\times \;10^{-12}$
Thymine	5.52 $\times \;10^{-13}$	2.01 $\times \;10^{-11}$	0.76–1.26 $\times \;10^{-11}$
Cytosine	1.47 $\times \;10^{-13}$	-	0.70–1.13 $\times \;10^{-11}$
Uracil	7.59 $\times \;10^{-14}$	5.03 $\times \;10^{-12}$	0.75–1.08 $\times \;10^{-11}$

**Figure A1:**
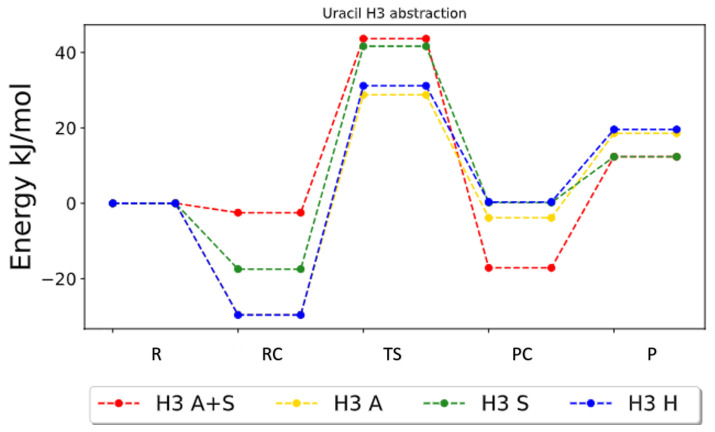
Zero-point vibrational corrected energies for the abstraction of H3 in uracil. A+S indicates anharmonic PCM calculation, A indicates anharmonic gas-phase calculations, S indicates harmonic PCM calculations and H indicales harmonic gas-phase calculations.

**Figure A2:**
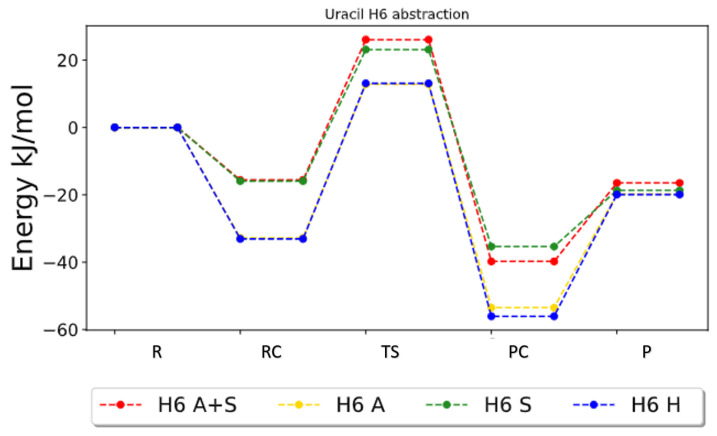
Zero-point vibrational corrected energies for the abstraction of H6 in uracil. A+S indicates anharmonic PCM calculation, A indicates anharmonic gas-phase calculations, S indicates harmonic PCM calculations and H indicales harmonic gas-phase calculations.

**Figure A3:**
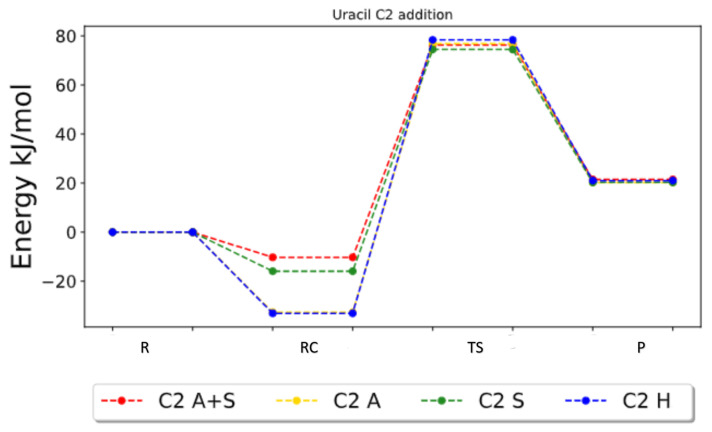
Zero-point vibrational corrected energies for the addition onto C2 in uracil. A+S indicates anharmonic PCM calculation, A indicates anharmonic gas-phase calculations, S indicates harmonic PCM calculations and H indicales harmonic gas-phase calculations.

**Figure A4:**
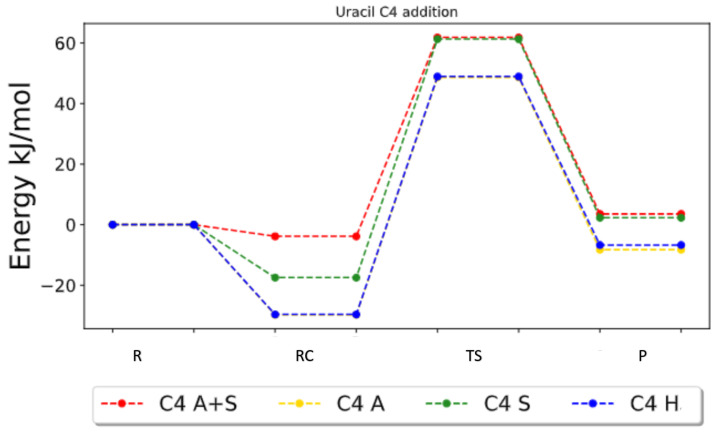
Zero-point vibrational corrected energies for the addition onto C4 in uracil. A+S indicates anharmonic PCM calculation, A indicates anharmonic gas-phase calculations, S indicates harmonic PCM calculations and H indicales harmonic gas-phase calculations.

**Figure A5:**
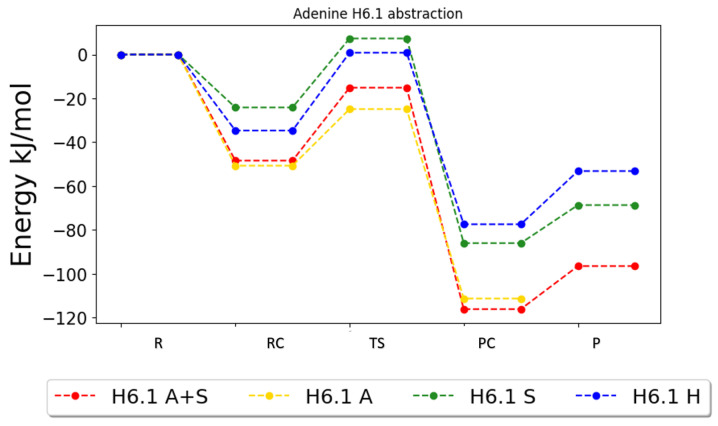
Zero-point vibrational corrected energies for the abstraction of H6.1 in adenine. A+S indicates anharmonic PCM calculation, A indicates anharmonic gas-phase calculations, S indicates harmonic PCM calculations and H indicales harmonic gas-phase calculations.

**Figure A6:**
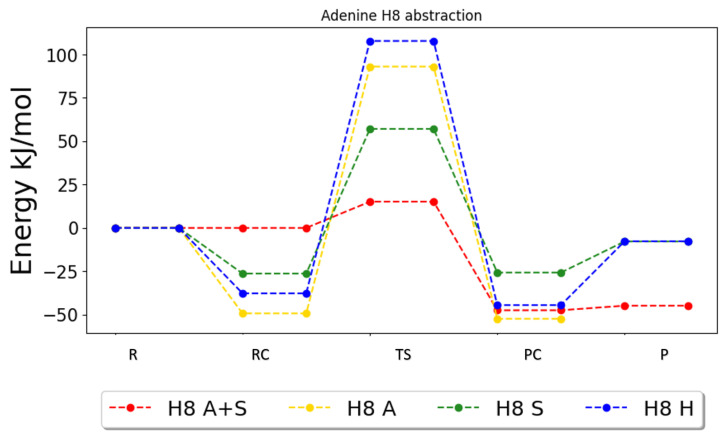
Zero-point vibrational corrected energies for the abstraction of H8 in adenine. A+S indicates anharmonic PCM calculation, A indicates anharmonic gas-phase calculations, S indicates harmonic PCM calculations and H indicales harmonic gas-phase calculations.

**Figure A7:**
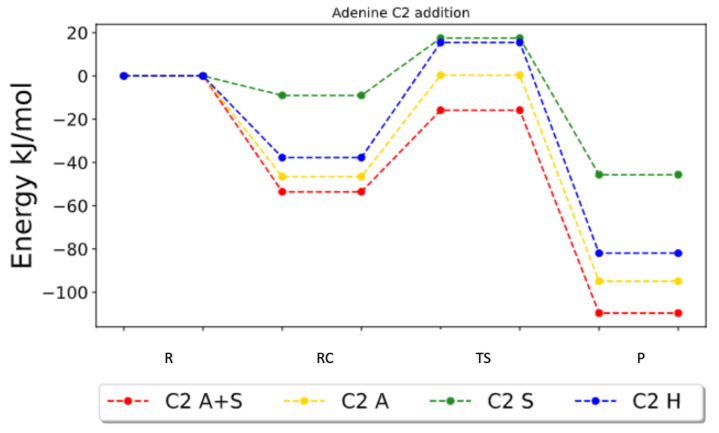
Zero-point vibrational corrected energies for the addition onto C2 in adenine. A+S indicates anharmonic PCM calculation, A indicates anharmonic gas-phase calculations, S indicates harmonic PCM calculations and H indicales harmonic gas-phase calculations.

**Figure A8:**
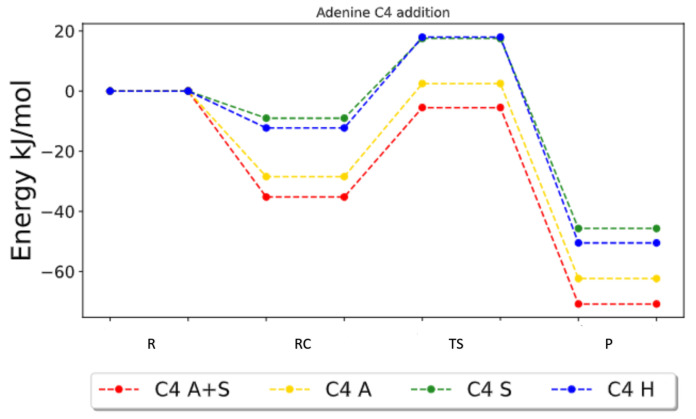
Zero-point vibrational corrected energies for the addition onto C4 in adenine. A+S indicates anharmonic PCM calculation, A indicates anharmonic gas-phase calculations, S indicates harmonic PCM calculations and H indicales harmonic gas-phase calculations.

**Figure A9:**
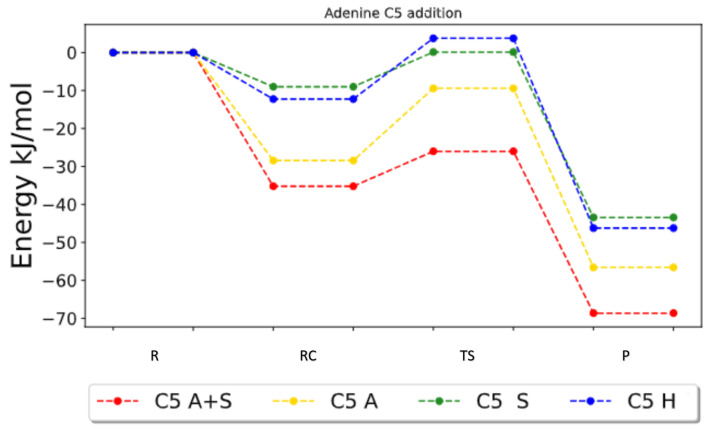
Zero-point vibrational corrected energies for the addition onto C5 in adenine. A+S indicates anharmonic PCM calculation, A indicates anharmonic gas-phase calculations, S indicates harmonic PCM calculations and H indicales harmonic gas-phase calculations.

**Figure A10:**
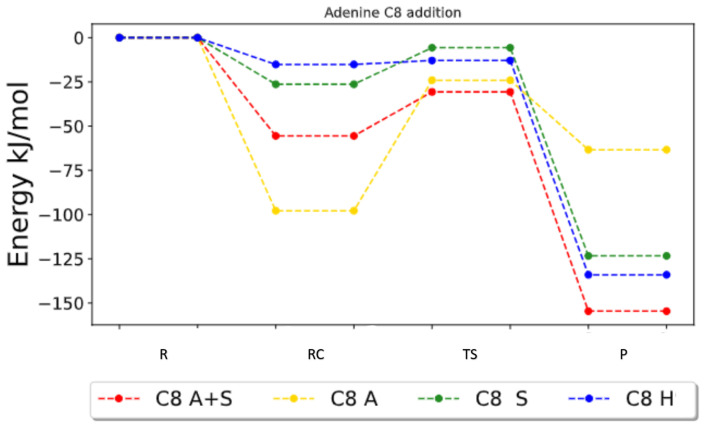
Zero-point vibrational corrected energies for the addition onto C8 in adenine. A+S indicates anharmonic PCM calculation, A indicates anharmonic gas-phase calculations, S indicates harmonic PCM calculations and H indicales harmonic gas-phase calculations.

## Data Availability

The data that support the findings of this study are available within the article. Further data are available from the corresponding author upon reasonable request.

## Abbreviations

The following abbreviations are used in this manuscript:

CPCM	Conductor-like Polarizable Continuum Model
DFT	Density Functional Theory
DNA	Deoxyribonucleic Acid
HOMO	Highest Occupied Molecular Orbital
IEF	Integral Equation Formalism
IRC	Intrinsic Reaction Coordinate
P	Product
PC	Product Complex
PES	Potential Energy Surface
PCM	Polarizable Continuum Model
R	Reactant
RC	Reactant Complex
RNA	Ribonucleic Acid
TS	Transition State
TST	Transition State Theory
